# Determination of the indoor radon concentration in schools of Tenerife (Canary Islands): a comparative study

**DOI:** 10.1007/s11869-022-01186-z

**Published:** 2022-03-29

**Authors:** María López-Pérez, Francisco Hernández, Juan Pedro Díaz, Pedro A. Salazar-Carballo

**Affiliations:** 1 Laboratorio de Física Médica y Radioactividad Ambiental, SEGAI, Universidad de La Laguna, San Cristóbal de La Laguna, Spain; 2 Grupo de Observación de La Tierra y La Atmósfera, Universidad de La Laguna, San Cristóbal de La Laguna, Spain; 3 Departamento de Medicina Física y Farmacología, Universidad de La Laguna, San Cristóbal de La Laguna, Spain

**Keywords:** Radon, Schools, Effective dose, COVID-19, Radioprotection

## Abstract

A radon survey was carried out in 18 high schools located in Tenerife Island when anti-pandemic strategies were used to reduce COVID-19 dissemination during 2021. High schools were located in radon-prone areas previously identified by the Spanish Nuclear Safety Council. Our results showed that 12 high schools presented radon activities lower than 100 Bq/m^3^, 5 high schools presented values in the range 100–200 Bq/m^3^, and only 1 high school presented radon activity concentration higher than 200 Bq/m^3^. Such values are below the reference level (300 Bq/m^3^) recommended by the Spanish legislation in the Basics Document of Health Standards (section HS6) of the Technical Building Code and the European Union directive (2013/59/EURATOM). Assuming an indoor occupancy time of about 1620 h per year, the annual dose contribution due to indoor radon exposure ranged from 0.07 to 1.18 mSv/year. Comparing such result against previous values reported in the literature on the island of Tenerife, we conclude that during the pandemic situation the indoor radon concentration (median valued) was reduced from 130.9 (2007) to 73.5 (2021) Bq/m^3^. Finally, continuous indoor radon concentration measurements were obtained to study short-time fluctuations (intra-day changes) under different ventilation conditions.

## Introduction

Radon (^222^Rn) is a natural radioactive gas that originated in the terrestrial crust and was released from the normal decay of the uranium (^238^U) primordial series. Radon was declared a human carcinogen by the US Environmental Protection Agency (EPA) in 1987 and by the International Agency for Research on Cancer (IARC) in 1988 (Fernández de Aldecoa 2000). Radon is the second leading cause of lung cancer after tobacco. In addition, most cases of radon-induced lung cancer occur in smokers, due to the strong synergic effect of tobacco and radon (Zeeb et al. 2009). In Europe, radon is responsible for 9% of deaths from lung cancer (Darby et al. 2005), and in Spain, it is estimated to be about 1500 deaths annually. Due to it being an inert gas, radon is free in nature and has great mobility within the earth’s crust, migrating from the place of origin (emanation) through pores and cracks in the lithosphere, and passing into the atmospheric air (exhalation) where it is dispersed. In addition, radon also may be generated, to a lesser extension, from the materials used for the construction of buildings, dwellings, etc. Outdoor radon concentration varies in the range of 5–15 Bq/m^3^. However, in indoor ambience such as homes, schools, offices, caves, mines, and basements, radon concentrations are higher, especially in spaces with minimal ventilation, reaching in some cases values up to 10,000 Bq/m^3^.

The indoor radon concentration depends on several factors. Firstly, and the most important factor, is the local geology: uranium/radium content, porosity, and permeability of the underlying bedrocks and soils. Secondly, the indoor radon concentration is not constant in buildings, caves, mines, etc., presenting intra-day and seasonal variations. Such behavior is driven by local meteorological variables such as temperature, pressure, humidity, wind speed, and rain. Thirdly, indoor radon concentration depends on the ventilation methods, habits of the occupants, construction materials, and the air tightness of the building. For example, exhaust fans can increase the indoor radon concentrations if adequate air intake is not provided, as they can produce negative indoor pressure and hence increase the radon exhalation. Finally, in most cases, passive systems of mitigation, such as improving the natural ventilation of the building, can reduce the indoor radon levels by more than 50%.

Because of these results, the European Commission published the new Directive (2013), obliging its members to update their legislation and establish the basic safety standards to protect the population from the risks of exposure to ionizing radiation. The new Directive sets the reference level for the average annual indoor radon concentration at 300 Bq/m^3^. Therefore, the Spanish Council published the Royal Decree (2019), amended the previous Technical Building Code (TBC), and included this reference value in the Spanish normative. Moreover, the Spanish Nuclear Safety Council (CSN), in its Safety Guide 11.2, establishes 300 Bq/m^3^ as the reference level; however, it recommends simple and economical remediation measures (for example, ventilation) from concentrations above 100 Bq/m^3^. On the other hand, for new buildings, the CSN also recommends a design level of 100 Bq/m^3^ (annual average of radon) (López-Pérez et al. 2020).

Radon exposure at schools has a substantial public health influence, affecting schoolchildren and school staff. However, the risk of lung cancer in children due to radon exposure may be up to threefold higher than in adults (Gordon et al. 2018). This is the result of lung morphometric differences (physiologic differences in shape and size) between children and adults and the higher respiration rates of children (increasing the radon exposition) (Gordon et al. 2018; Kojo and Kurttio 2020; Martin et al. 2020). In addition, children also spend more time indoors. It can be estimated that Spanish children, on average, may spend 1620 h per year in school buildings. Nevertheless, this estimation does not include additional hours that children might spend in after-school programs.

In 2020, during the COVID-19 pandemic, the Spanish Council and other governments (Minguillón et al. 2020) and international agencies recommended multiple preventive actions, including improvements to building ventilation, to reduce the risk of exposure and the spread of disease (Stabile et al. 2021; Ziauddeen et al. 2020; Zivelonghi and Lai 2021). Therefore, it was recommended to improve the ventilation condition of the classroom with easy and economical methods such as open windows and doors, when weather conditions allow, to increase the outdoor airflow. This situation opens the possibility to study and compare how radon level exposure can be affected in schools during this special pandemic situation. In Canary Island, few works have been developed in this area; to the best of our knowledge, only a previous work, reported by our group in 2007, is found in the literature (Hernández et al. 2007). In this previous work, the radon exposure was calculated using Makrofol detectors, and after studying the radon concentration in 62 sampling sites, we estimated that the mean indoor radon concentration in the school of Tenerife was about 132 Bq/m^3^.

## Materials and methods

### Geological setting

**Fig. 1:**
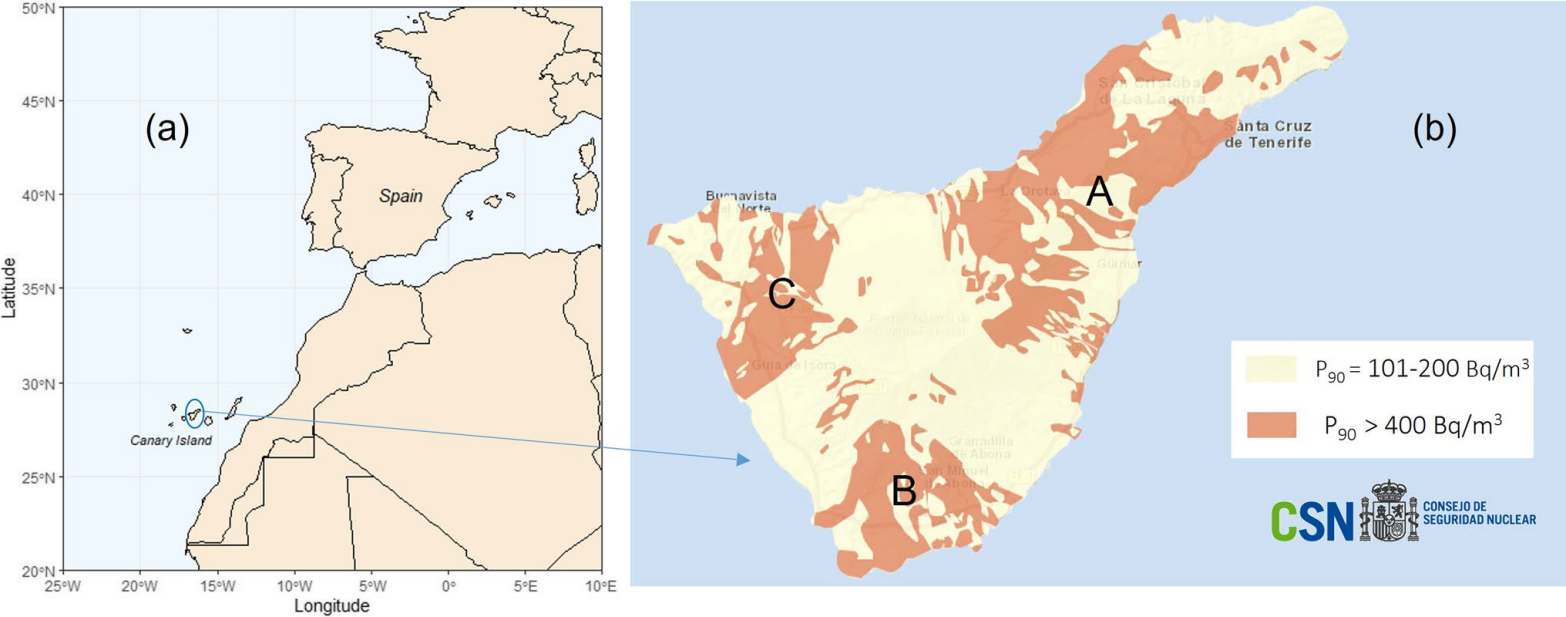
**a** Geographic location of the Canary Islands. **b** Radon potential map of Tenerife Island. P_90_ represents that 90% of the buildings have concentrations below the reference value and 10% exceed this level. Source: Spanish Nuclear Safety Council (CSN), https://www.csn.es/mapa-del-potencial-de-radon-en-espana

The present study was carried out on the island of Tenerife, in the Canary Islands. The Canary Islands are a volcanically active archipelago formed by eight main islands. It covers an area of 7447 km^2^ and is located northwest of the African coast, between 27° 37′–29° 25′ N latitude and 13° 20′–18° 10′ W longitude (see Fig. 1a). Tenerife is the largest island of the Canarian archipelago (with an area of ca. 2034 km^2^) and occupies a central position in the archipelago. The main geological features of Tenerife are three main volcano-tectonic rifts trending N-E, N-W, and N-S and, at the interception center of these three volcano-tectonic axes, Las Cañadas caldera and the stratovolcanoes Teide-Pico Viejo. Due to its volcanic origin, and especially due to the different types of eruptive materials generated (from non-differentiated mafic to differentiated felsic materials), uranium and its progeny can be found in different concentrations along its surface. In addition, due to recent volcanism events and remodeling processes (soil alteration, erosion, land-slide episodes, etc.), different materials are exposed on its surface. From a radiological point of view, ultra-basic and basic rocks such as nephelinites, basanites, and trachy-basalts contain low ^40^ K, ^226^Ra, ^232^Th, and ^238^U concentrations. However, intermediate and felsic rocks such as trachyandesite, basaltic-andesite, andesite, trachytes, phonolites, and peralkaline rhyolites contain higher concentrations of radioactive elements (Faure 2005). From the radiological point of view, the reader may find more information in previous works of the group (López-Pérez et al. 2021). In the last work, terrestrial gamma absorbed dose rates as well as the spatial distribution of gamma-emitting radionuclides ^40^ K, ^226^Ra, and ^232^Th and ^137^Cs in soils of the Western Canary Islands were presented.

### Epidemiologic studies and radon-prone areas

Radon-prone areas in the Canary Island and mainland Spain areas have been identified by the CSN (Evangeliou et al. 2013). Such areas are defined as areas with the probability of finding high indoor radon concentrations. According to such a definition, 19% of the Canarian territory is classified with high radon risk (Burger and Lichtscheidl 2018). Nevertheless, such areas are identified in Gran Canaria and Tenerife, where ca. 84% of the inhabitants of Canary Island are found. In Fig. 1b, P90 (percentile-90) distribution of radon is represented for Tenerife Island, where ca. 45% of the territory is cataloged as a radon-prone area. P_90_ represents areas where 10% of the indoor radon measurements may be higher than a fixed reference level. Moreover, in previous epidemiologic works (Ruano-Ravina et al. 2021), the radon exposition by autonomous regions (AR) in the Spanish territory was computed. Such work concluded that the main AR affected, with the highest population percentages exposed to high radon concentrations (> 300 Bq/m^3^), and after dwelling height correction, were Galicia (7.0%), Extremadura (6.9%), and Canary Islands (5.5%). This study showed that 3.8% (838 deaths) of lung cancer mortality in Spain was attributable to residential radon, being most of lung cancer deaths in smokers and ex-smokers.

### Methodology

This study was conducted in 18 high schools located on Tenerife Island during the rainy season. The distribution of such high schools was according to the radon-prone areas delimited by the CSN (Jan 1996) and taking into consideration the number of inhabitants in these three areas (A, B, C) (for details, see Fig. 1b). Therefore, 11 high schools were sampled in Zone A (the most habited zone), 6 high schools in Zone B, and 1 high school in Zone C. To this end, nineteen CR-39 passive detectors were exposed (five in each center) for a period of ca. 90 days, mainly in classrooms and some offices, libraries, and study rooms (spaces mainly used by students, teachers, and staff). Such passive detectors were selected because they are sensitive to a range of energy from 0.1 to 40 MeV, ideal for alpha particle measurements. During this period, the classrooms and studied spaces were normally used, and according to the new ventilation actions recommended during the pandemic situation. Detectors were located at a height of about 1–1.5 m above the floor as representative of breathing height, avoiding sites with excessive ventilation, closed lockers, heat source, and airstream, and avoiding the summer months to mitigate the seasonal variations (as recommended by CSN and TBC). Moreover, in high schools with two or more floors, the ground floors were sampled with a higher number of detectors, and the number of the floor, ventilation conditions, and the number of pupils were registered. Finally, the detector distribution, according to the level floor, was − 1 (14 detectors), 0 (40 detectors), 1 (30 detectors), and 2 (6 detectors). In addition, for divulgation purposes and during the sampling period, several oral expositions were done in the high schools entitled “*Radon in Schools*” to introduce radon, its radiological properties, and common mitigation method for reducing its exposure. After exposure periods (ca. 3 months), every detector was retrieved and re-sealed to avoid continued exposure and sent immediately to an accredited laboratory according to UNE-EN ISO/IEC 17025:2005 for integrated radon air measurements with CR-39. In some cases, when the results obtained during the analysis were lower than the limit of detection (LOD) of the method, such a value was assigned for this measure for conservative purposes.

The annual mean effective dose (*H*_Rn_) received due to radon gas exposure was estimated from the concentration obtained during the 3 months, and assuming that this value was equal to the annual mean value. *H*_Rn_ was calculated according to UNSCEAR recommendation (Kawada and Yamada 2012) as

1 $$H (mSv/year)=CRn\times F\times O\times DCF$$

where *C*_Rn_ = indoor radon activity concentration [Bq/m^3^], and *F* is the equilibrium factor between radon and its decay products (0.4). *O* is the average indoor occupancy time per pupil or teacher (1620 h per year), and DCF is the dose conversion factor for radon exposure (9 · 10^−6^) [mSv · m^3^/Bq · h] (Lecomte et al. 2014).

To better understand how intra-day variations are affected in different scenarios, continuous indoor radon activity concentration was monitored using the Radon Scout Plus detector (SARAD GmbH) in different classrooms at the Faculty of Health Science of the University of La Laguna. Such a detector is a portable equipment with a solid-state detector (Silicon) designed for the integrated measurement of gross alpha activity. It is a detector with small dimensions, very light, without mechanical parts such as membrane pumps or an external power supply that can autonomously work with two batteries of 1.5 V. In addition, it can register other ambient parameters such as air temperature, relative humidity, and barometric pressure. Due to it works under diffusion conditions, such detector does not cause any disturbance or annoyance to the students during the measurement process.

## Results and discussions

### Radon survey during preventive actions for reducing COVID-19 pandemic

**Fig. 2:**
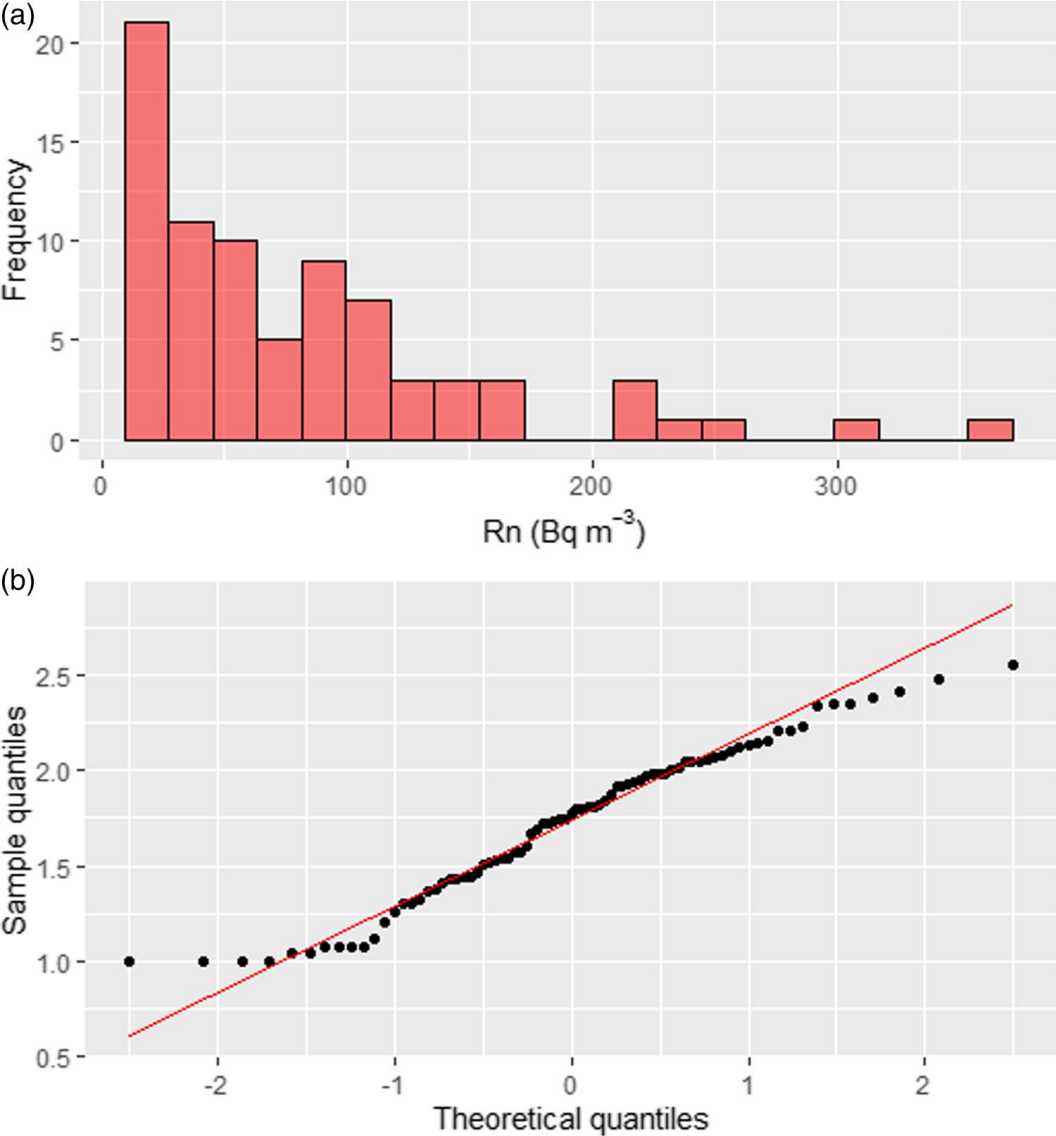
**a** Histogram of the radon concentrations survey performed in 79 sample points in 18 high schools of Tenerife Island. **b** Quantile–quantile plot for the log-transformed data

A total of 90 alpha-track radon detectors were installed during ca. 3 months in different high schools of Tenerife Island. After finishing the exposition time, they were retrieved to be analyzed; however, only 79 detectors (88%) could be found. The final floor distribution of the recovered detectors was as follows: − 1 (10 detectors), 0 (36 detectors), 1 (27 detectors), 2 (6 detectors). Figure 2 shows the histogram and the quantile–quantile plot of our data. As expected, data are well-defined by a log-normal distribution, typical for many geological data. Such a distribution is characterized by a skew distribution with many small values and fewer large values. Thus, the mean value of the data is usually greater than the mode, and it is not suitable for comparison purposes because the mean value is influenced by extreme values. The Kolmogorov–Smirnov test with Lilliefors corrections of the original data (*p* value < 0.05) and the log-transformed data (*p* value = 0.2) confirmed such affirmation. Analyzing the data distribution, we found that 56 detectors (70.9%) registered radon activities lower than 100 Bq/m^3^, 21 (26.6%) presented values in the range 100–300 Bq/m^3^, and only 2 (2.5%) detectors were exposed to radon activities higher than 300 Bq/m^3^.

**Fig. 3:**
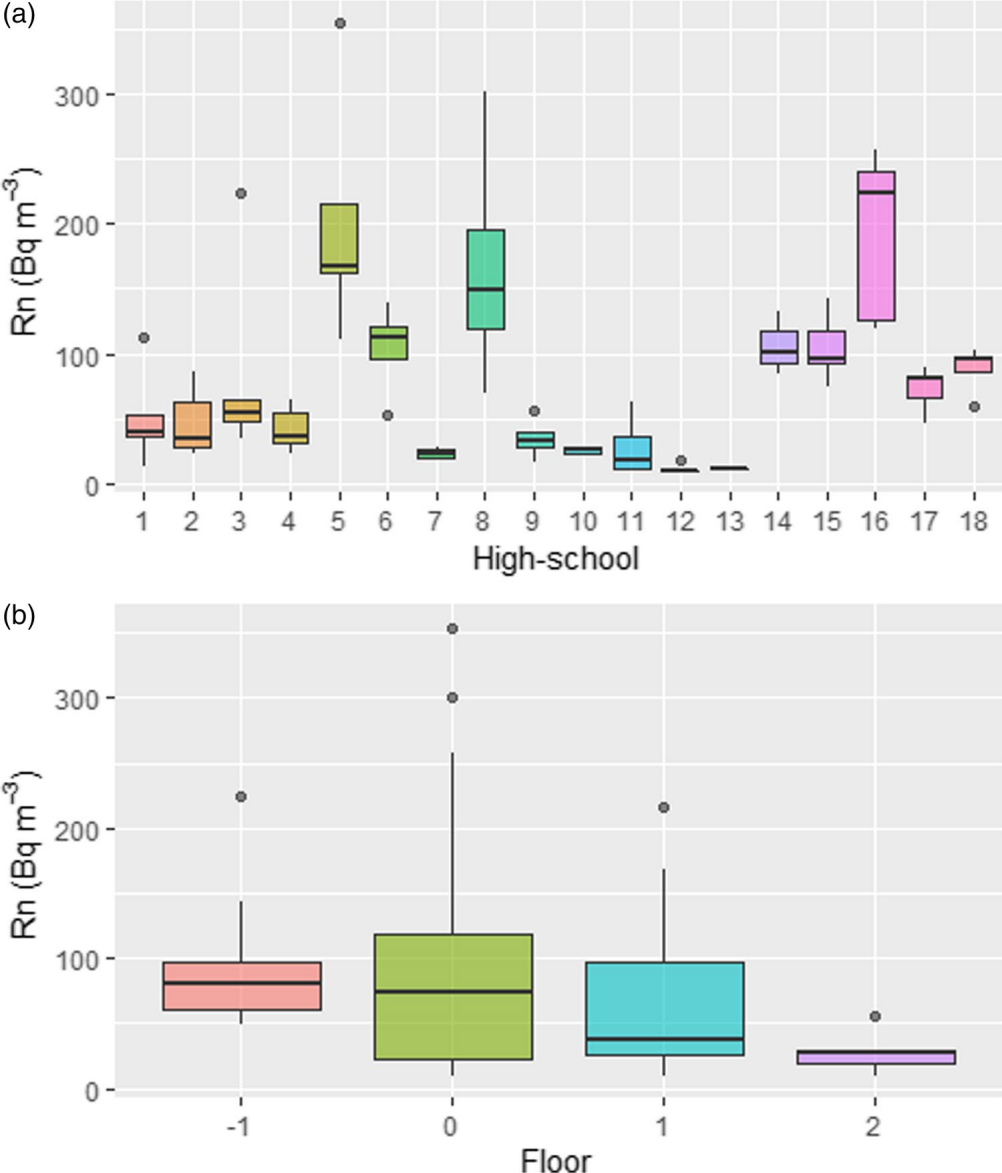
**a** Box-and-whisker plots of the radon concentration survey performed in 79 sample points in 18 high schools of Tenerife Island. **b** Box-and-whisker plots for the radon values grouped by the floor level

Table 1 and Fig. 3a show the main results obtained for each high school. The mean value and standard deviation of the recovered radon detectors were calculated according to the TBC recommendations. Such data showed that 12 high schools presented radon activities lower than 100 Bq/m^3^, 5 high schools presented values in the range 100–200 Bq/m^3^, and only 1 high school presented radon activity concentration higher than 200 Bq/m^3^. According to Eq. 1, the annual mean effective dose attributable to radon exposure was calculated (*H*_Rn_). In addition, the percent of dose compared against the worldwide average natural dose (2.4 mSv/year), reported by the UNSCEAR (Lecomte et al. 2014), was computed. It can be observed that *H*_Rn_ (see Table 1) values ranged from 0.07 to 1.18 mSv/year. The annual dose contribution due to radon exposure in the high schools of Tenerife Island was calculated about 20% of the annual doses attributable to the total ionizing radiation, with some extreme values higher than 40% in high schools numbered as 5, 8, and 16.

**Table 1:** Results of the radon concentration measurements carried out in the different schools of Tenerife Island

High school	Zone	Mean (Bq/m^3^)	Stand. dev	*n*	*H*_Rn_ (mSv/year)	% of the WAND
1	**B**	51.0	37.0	5	0.30	12.4
2	**B**	47.0	26.7	5	0.27	11.4
3	**A**	85.6	78.1	5	0.50	20.8
4	**B**	42.4	16.7	5	0.25	10.3
5	**A**	202.2	92.7	5	1.18	49.1
6	**A**	104.3	36.4	4	0.61	25.3
7	**B**	23.5	4.1	4	0.14	5.7
8	**B**	177.0	116.8	4	1.03	43.0
9	**A**	34.8	16.4	4	0.20	8.4
10	**A**	25.7	4.2	3	0.15	6.2
11	**C**	28.0	24.5	4	0.16	6.8
12	**A**	11.6	3.6	5	0.07	2.8
13	**A**	12	0.0	4	0.07	2.9
14	**B**	106	24.4	3	0.62	25.8
15	**A**	104.8	26	5	0.61	25.5
16	**A**	193.4	65.4	5	1.13	47.0
17	**A**	73.6	17.3	5	0.43	17.9
18	**A**	88.5	19.2	4	0.52	21.5

*WAND* worldwide average natural dose

Figure 3b displays the radon activity results against the floor number. The indoor radon concentrations (arithmetic mean) were 94.8, 95.4, 64.4, and 27.7 Bq/m^3^ for the basement, ground floor, and first and second floors respectively. A clear trend can be observed with lower activity value in the higher floors. However, the nonparametric Kruskal–Wallis *H* test was used to determine if there were statistically significant differences between the different floor levels. Such a test is considered the nonparametric alternative to the one-way analysis of variance (ANOVA) and an extension of the Mann–Whitney *U* test (a nonparametric test used to compare only two independent groups). The Kruskal–Wallis *H* test showed that there was a statistically significant difference in radon exposure between the different floor levels (*χ*^2^ = 8.038, *p* = 0.04) with a mean rank radon activity of 50.6, 43.2, 36.3, and 20.1 Bq/m^3^ for the basement, ground floor, and first and second floors, respectively.

Indoor radon concentration ratios by storey level were obtained. Therefore, the basement/ground floor ratio, ground floor/first-floor ratio, and ground floor/second floor ratio were ca. 1, 1.5, and 3.4, respectively. The second one (ground floor/first-floor ratio) is well reported in the literature and ranged from 1.4 to 1.6, in good agreement with the value obtained in this work (Briones et al. 2021).

**Fig. 4:**
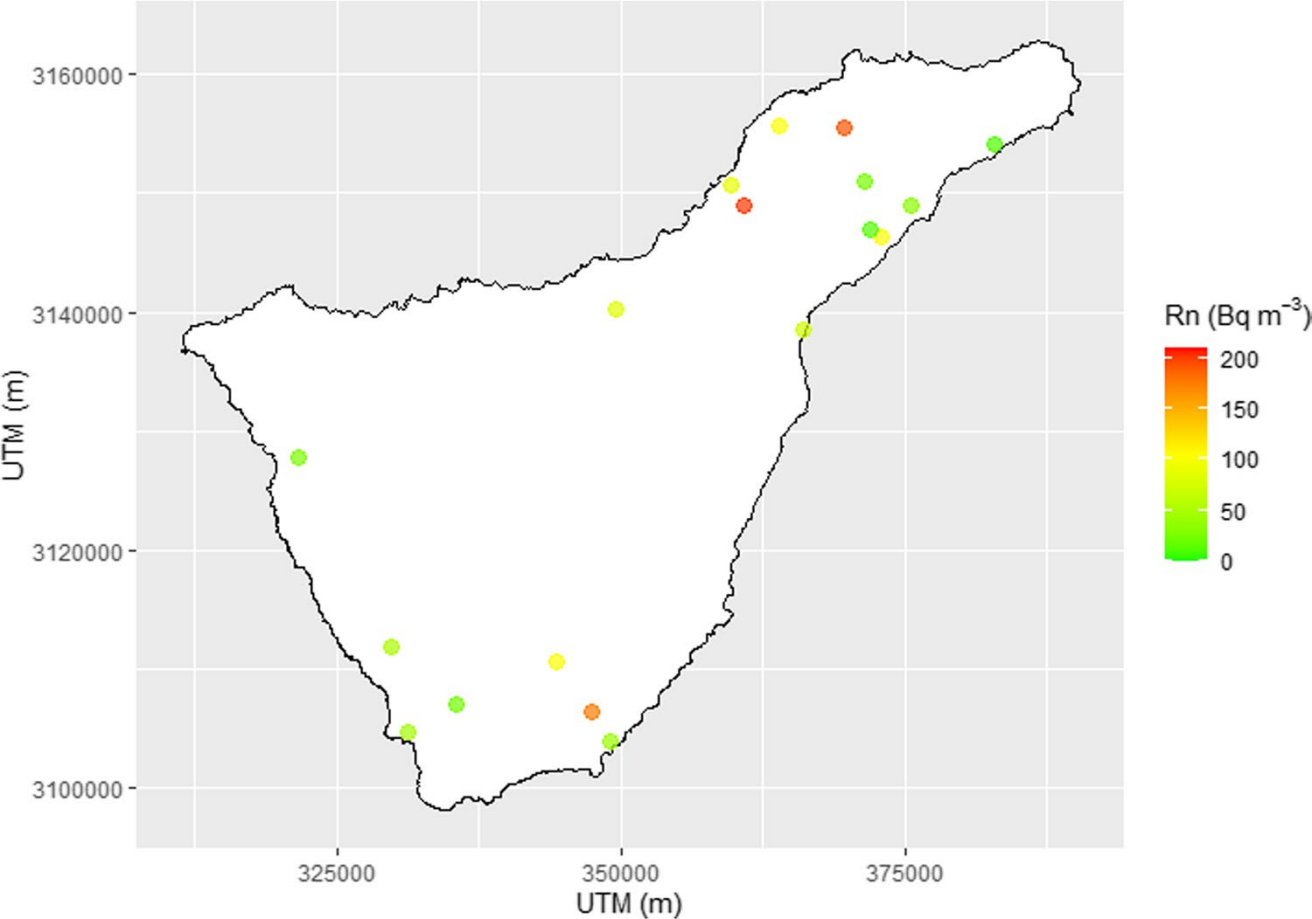
Distribution mean values of the radon activity concentration obtained in 18 high schools of Tenerife Island

The geography distribution of the mean radon activity is displayed in Fig. 4. However, the small number of sample points does not allow us to infer trends associated with the geology of the island. The different radon-prone zones (A, B, and C) were sampled in this study; however, zone C has only 1 localization due to problems recruiting participants. Nonparametric Mann–Whitney *U* test (nonparametric test equivalent to the two-sample independent *t* test for normal distribution) was used to check if a significant difference was found between radon activity concentration between the different radon-prone zones (A and B). Such analysis confirmed that the two distributions were not significantly different at 0.05 level of confidence (*z*-score = 0.67, *p* value = 0.25). Such analysis confirmed that there was no radon exposure difference between zone A and B.

### Comparative analysis for radon exposure before (2007) and during COVID-19 pandemic (2021)

In a previous radon survey developed by our group in 2007, different schools were studied to locate radon-prone zones on the island of Tenerife. The methodology of the previous survey was slightly different. On such occasions, 62 schools were sampled during the dry season. The sampling points, during this survey, were always situated in the basement of the schools. In addition, just one sampling point (by duplicate) was obtained for each school. The school distribution was also very similar, focusing their locations on the radon-prone areas identified in TBC and according to the population density. Due to the objective of the present section being to compare how the ventilation may affect the indoor radon activity in the classrooms, we directly use the values obtained in each studied classroom (79) during the dry season in 2021, not the mean value for each center. In addition, previous data (2007) were corrected, multiplying such values by a seasonal factor (1.4) (Al-Khateeb et al. 2017; Arvela et al. 2015; Otoo et al. 2018). Such correction is commonly used in the literature, and it is recommended in the TBC to calculate the annual indoor radon concentration when the radon measurements are obtained during the dry season. Usually, seasonal variations are characterized by higher indoor radon concentrations in winter than in summer. This behavior can be easily understood due to soil moisture and drying may change the transport properties of the soil air. Therefore, when the land surrounding a building is soaked during the winter, radon tends to enter the building through the dried basement, with greater permeability.

**Fig. 5:**
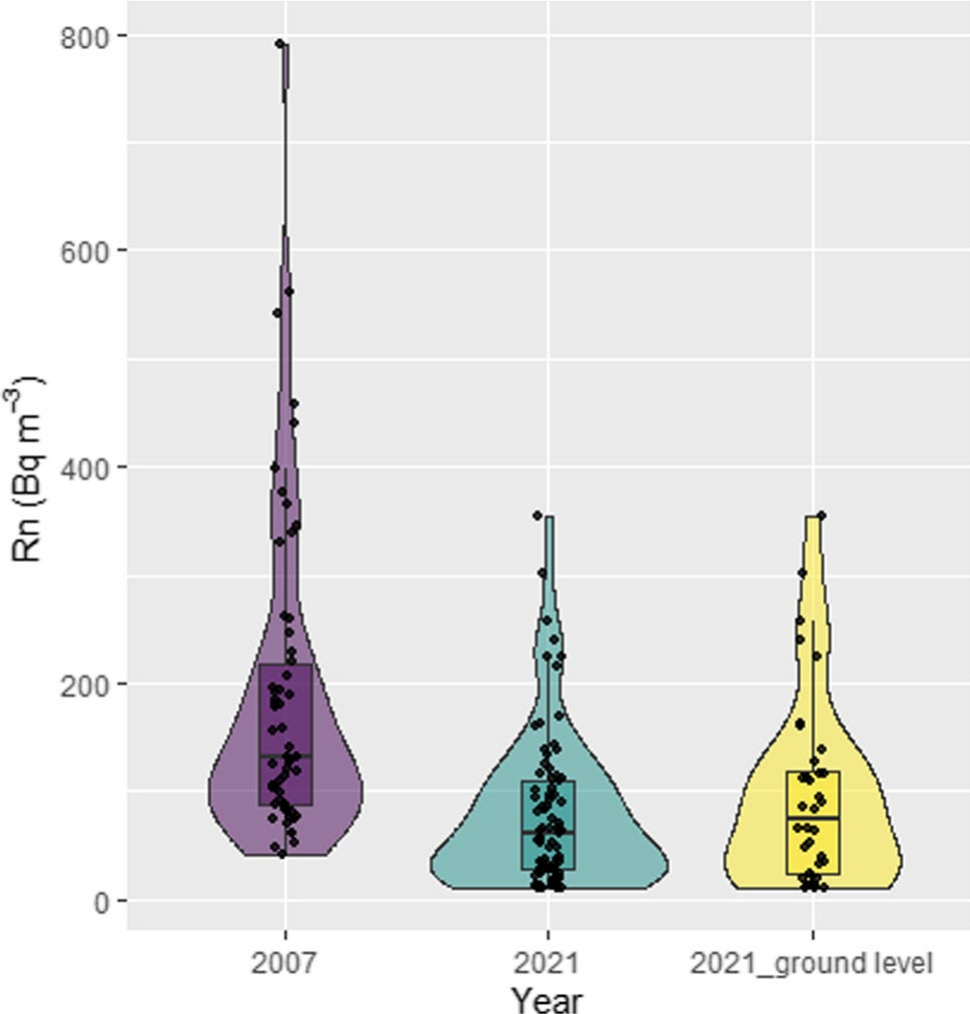
Box-and-whisker and violin plots of the radon concentration surveys developed in Tenerife Island before (2007) and under the COVID-19 pandemic (2021)

Figure 5 shows the box-and-whisker plots for the two surveys (2007 and 2021). Moreover, the ground floor data for 2021 are also shown. It is convenient to indicate that for the correct comparison between the two radon surveys, only ground values (during the 2021 survey) were included in this study. Such data selection was done to avoid underestimation during the analysis due to indoor radon concentration decrease with the storey level, and the previous radon survey (during 2007) was obtained on the ground floor in schools of Tenerife Island (see above). Box-and-whisker plots show that during 2021 it seems that the lowest values are presented in a greater proportion (for comparison purposes, see violin plots in Fig. 5). The median values registered were 130.9 and 73.5 Bq/m^3^ for 2007 and 2021 (ground level) respectively. Therefore, such analysis points out that during 2021, when anti-COVID-19 strategies were used to reduce pandemic dissemination, the radon exposure in the high school of Tenerife Island was reduced. Newly, the nonparametric Mann–Whitney *U* test was used to check if a significant difference was found between radon exposure between 2007 and 2021. Such analysis confirmed that the two distributions were significantly different (*z*-score = 3.84, *p* value < 0.05) and confirmed that during the period when COVID-19 pandemic actions to decrease the virus transmission were applied, indoor radon exposure in schools was also reduced.

### Intra-day and other indoor radon concentration fluctuations

**Fig. 6:**
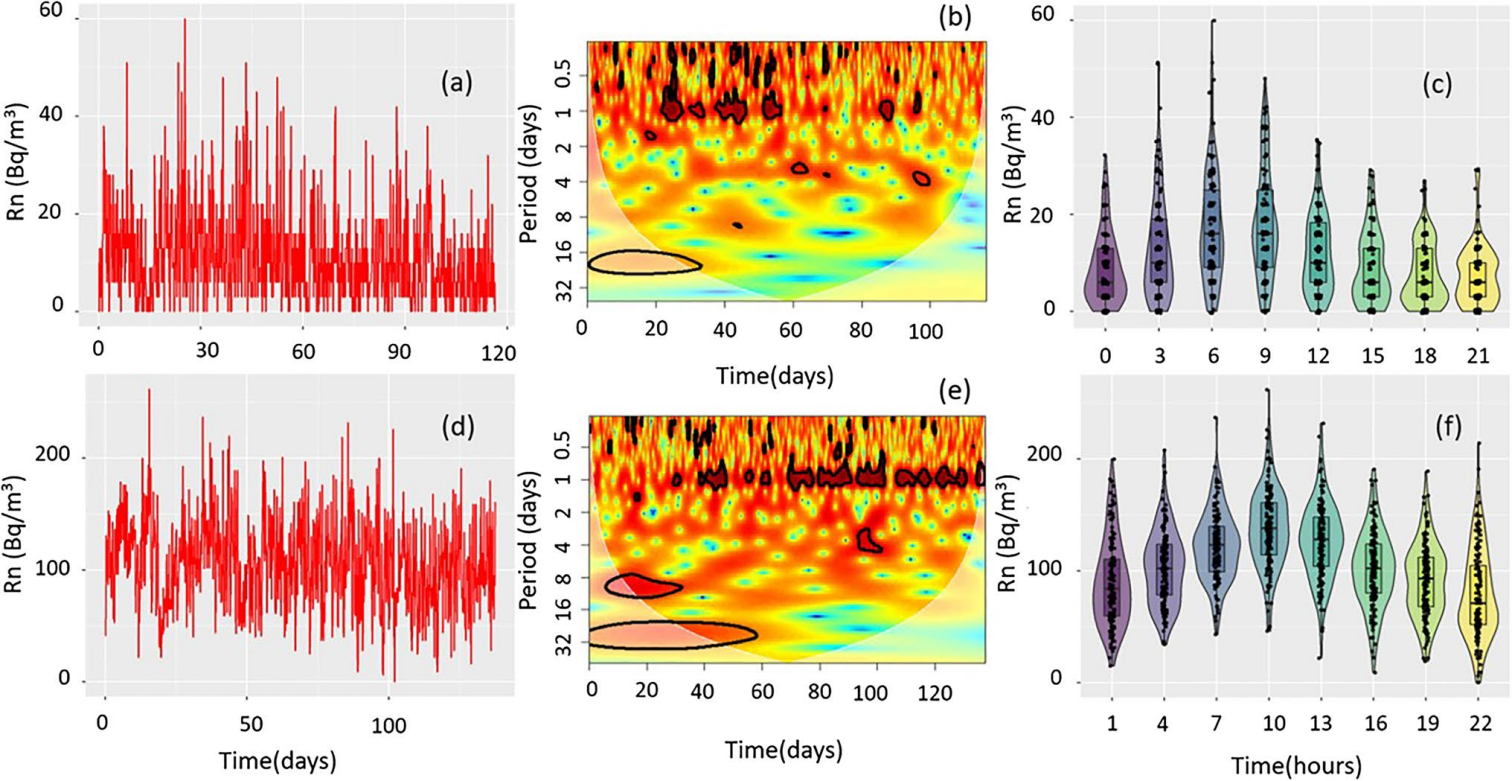
Radon indoor concentration time series obtained during ca. 3 months in classrooms located in a second (**a**) and underground floor (**d**). Continuous wavelet transforms for data registered in classrooms located in a second (**b**) and underground floor (**e**). Box-and-whisker plots for data registered in classrooms located in a second (**c**) and underground floor (**f**)

Indoor radon concentration in buildings is affected by many physical factors and processes, including some constant effects such as radionuclide concentration of the bedrock and nature of the construction materials. However, other ones may change within the time such as soil moisture, temperature, atmospheric pressure, and ventilation methods. Therefore, the indoor radon concentration usually presents variation at different temporal scales (seasonal, weekly, intra-day fluctuations, etc.). For radiological protection applications, such sources of variation may play a crucial role to understand and to calculate the correct dose received by the inhabitants, workers, etc. For example, continuous monitoring radon devices can register the radon concentration each hour, and the indoor radon exposure can be calculated only by taking into consideration the teaching hours. In addition, such an approach allows us to obtain a better comparison about the ventilation methods used for mitigation purposes and their efficiency to reduce indoor radon exposure. Figure 6a and d display the time series for two classrooms at different storey levels. Classrooms were located on a second and underground floor; the last one had poor ventilation. Radon levels for these two classrooms were 11.7 ± 9.8 (second floor) and 106.4 ± 39.8 Bq/m^3^ (underground floor). Although the temporal variation of such time series seems to be random behavior, a deep analysis in the time or frequency domain may help to understand their temporal structure. In this regard, the continuous wavelet transform (CWT) may help us to decompose the time series into a time–frequency space with multi-time resolution. Figure 6b shows the CWT analysis for the radon time series located on the second floor. It can be observed that few cyclic events are detected in the daily frequency (the thick black contour indicates the 95% confidence level). Such findings may be justified due to the low radon concentration registered; the concentration may be random in nature. In addition, at these levels of concentration the instrumental error (> 20%) may cause important artifacts. On the other hand, the CWT for the ground floor (Fig. 6e) presented a clearer cyclicality (daily cycle) with some degree of intermittency at the beginning of the time series and other periodic contributions (weekly and monthly contributions). However, such low-frequency modulations are very close to the cone of influence (COI), the dashed region in the figure limited by a white line. COI evaluates scalogram areas (at the beginning and the end of the analyzed signal) potentially affected by edge-effect artifacts. Thus, to obtain a more robust analysis in the low frequencies, we need a longer time series.

On the other hand, the ventilation habits may help to reduce indoor radon exposure; however, only using a continuous radon analysis we may analyze if such remediation method is correctly applied. Figure 6c shows the box-and-whisker plot for the data obtained in the classroom located on the second floor. As observed, we can intuit that the higher radon concentration occurs during the first hours in the morning; however, due to the low radon level presented and a good ventilation method (opening windows and doors), such value is reduced. However, on the underground level (with poor ventilation), although mechanical ventilation is used, the radon level is still higher than 100 Bq/m^3^ during the teaching hours. Newly, during the night when the classrooms are closed and non-ventilated, the indoor radon concentration increases again. Therefore, such results may help us to design and improve new ventilation methods that allow obtaining the minimum indoor radon level during the teaching hours.

**Fig. 7:**
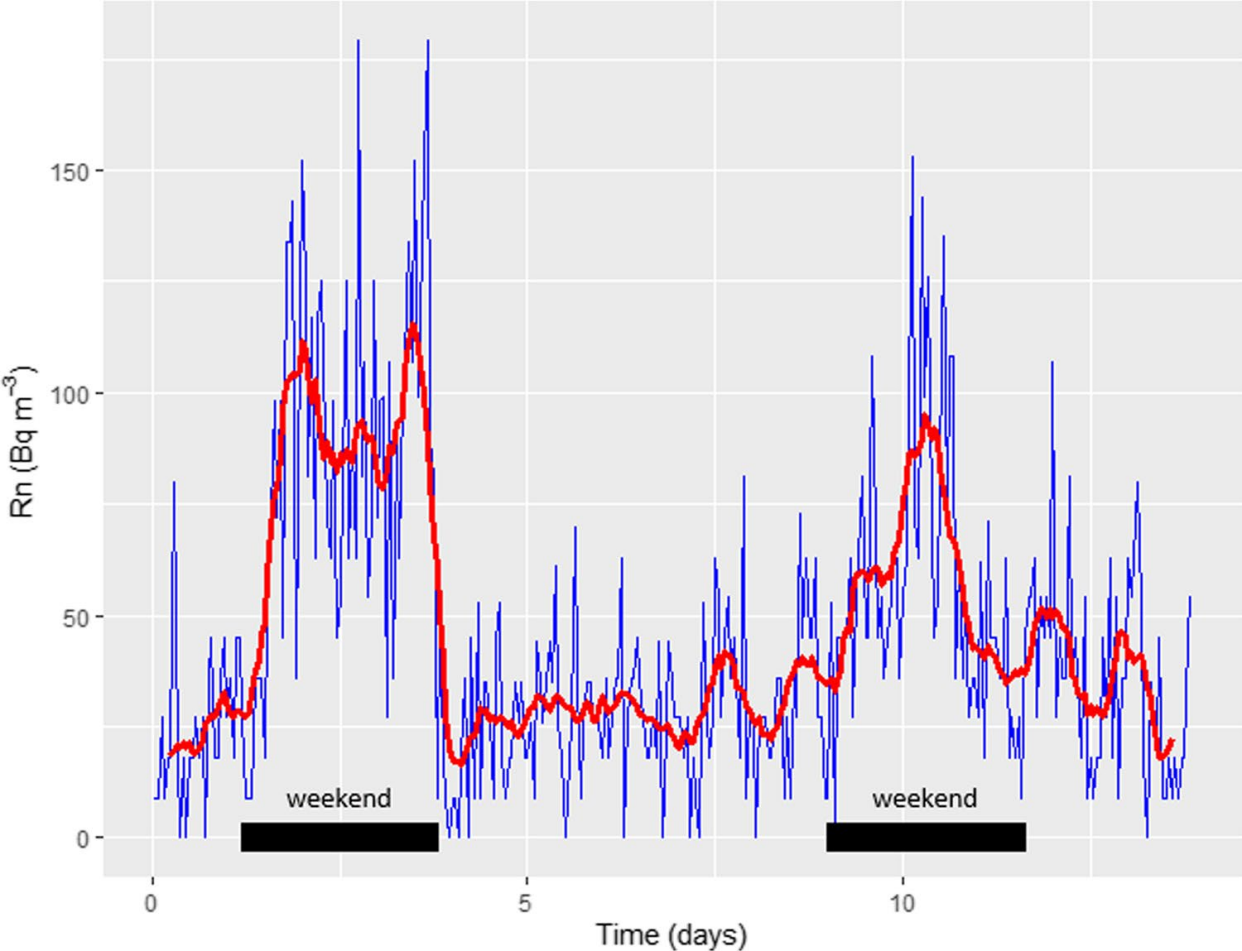
Indoor radon concentration time series obtained for 15 days in a classroom situated on the ground floor

Finally, other interesting results, obtained during the continuous monitoring of the indoor radon concentration, are shown in Fig. 7. Here, we can observe how the radon level increases during the weekend when natural ventilation is not used. It is important to remark that during COVID-19 pandemic mitigation actions many high schools kept the doors and windows open all the time and only during the weekend the classrooms were closed. Figure 7 displays how during the week the radon levels were below 50 Bq/m^3^; meanwhile, during the weekend (when doors and windows were closed) such levels increased until 150 Bq/m^3^. Such an effect can be obtained during other periods, for example, holidays, and may produce an overestimation in the indoor radon concentration and hence in the calculated dose received by pupils and teachers. For example, using these results, and calculating the median of the full data and without the 2 weekend periods, we calculate an overestimation of about 35%. Such results confirm that although passive detection used for long exposition is highly convenient and is recommended by the Spanish legislation, it should be used with some precaution. Therefore, in the TBC we can find several correction factors that take into consideration the seasonality changes during the dry and wet seasons and correction about the degree of ventilation and occupancy of the spaces. Of course, the best recommendation is to do normal use of the classroom, office, and home during the sampling period.

## Conclusions

In the present study, the indoor radon concentration in different high schools of Tenerife Island was obtained during COVID-19 pandemic (2021). Our results confirm that although such high schools are situated in radon-prone areas (identified by the Spanish Nuclear Safety Council), the indoor radon concentration does not represent a risk for the health of the student, teacher, and staff. Sixty-seven percent of the high schools presented values lower than 100 Bq/m^3^ and 22% below than 200 Bq/m^3^, and only one (5%) high school presented an indoor radon concentration (202 Bq/m^3^) slightly higher than 200 Bq/m^3^. We argue that these low values, contrasted with previous studies, may be attributable due to the extraordinary ventilation circumstances used during the COVID-19 pandemic in the classrooms.

## Data Availability

The datasets generated and/or analyzed during the current study are not publicly available but are available from the corresponding author on reasonable request.
